# Microglia-Neuron Interactions in Alzheimer’s Disease

**DOI:** 10.2174/011570159X379539250807114252

**Published:** 2025-08-26

**Authors:** Yujie Ma, Xinyue Wang, Minghuang Gao, Yeze Lin, Qini Chen, Hongyin Yang, Cong Yang, Qi Wang

**Affiliations:** 1 Science and Technology Innovation Center, Guangzhou University of Chinese Medicine, Guangzhou, 510405, China;; 2 Department of Oncology, The Sixth Affiliated Hospital, Sun Yat-sen University, Guangzhou, 510655, China;; 3 Guangdong Provincial Key Laboratory of Colorectal and Pelvic Floor Disease, Guangdong Research Institute of Gastroenterology, The Sixth Affiliated Hospital, Sun Yat-sen University, Guangzhou, 510655, China

**Keywords:** Alzheimer’s disease, microglia, neuronal degeneration, synaptic pruning, metabolic coupling, classical hypothesis

## Abstract

Alzheimer's disease (AD) is a progressive disease characterized by significant cognitive decline, posing a substantial threat to life. Neuronal loss and dysfunction are responsible for the cognitive decline and behavioral disturbances observed in AD. Microglia are increasingly recognized for shaping the fate of neurons. However, the role of microglia-neuron interaction in neuronal degeneration of AD remains largely unclear. This review discusses microglia-mediated excessive synaptic pruning and microglia-neuron metabolic coupling in the neuronal degeneration of AD. It also summarizes the role of microglia-neuron interactions in classical pathogenic hypotheses such as the amyloid cascade, tau protein, neuroinflammation, and metal ions. It is found that microglia can serve as protectors of neurons, yet they also exacerbate neuronal damage under stress stimulation. This bidirectional modulation of microglia-neuron interaction provides a novel direction for rescuing AD neurons.

## 1 INTRODUCTION

More than a century has elapsed since the initial report of Alzheimer's disease (AD). The prevalence of AD continues to escalate rapidly, positioning it as the sixth leading cause of death in the United States [1]. The swift aging of the global population further amplifies AD's threat to human health. Numerous studies have concentrated on two primary pathological features of AD: amyloid β (Aβ) deposition and tau hyperphosphorylation. However, despite extensive research efforts, no significant breakthroughs in treatment strategies have emerged. Although phase III clinical trials for clecanemab-a monoclonal antibody targeting Aβ-have demonstrated its benefits, controversy persists regarding its efficacy [2]. Furthermore, it remains uncertain whether current therapeutic approaches can effectively mitigate the public health burden associated with AD [3].

The progressive loss and dysfunction of synapses and neurons are hallmark characteristics of AD. Synapses serve as critical units for signal processing in the brain and form the basis for neural plasticity [4, 5]. They release synaptic vesicles in response to action potentials to convey information, thereby establishing neural circuits essential for learning, memory, cognition, emotions, movements, and skills [6, 7]. Typical clinical manifestations of AD include memory impairment, cognitive deficits, and behavioral disturbances-all resulting from neuronal damage [8, 9]. An initial period of overactivity often precedes neuronal loss; this disrupts synaptic homeostasis and correlates with subsequent neuronal death [10]. Evidence suggests that enhancing synaptic plasticity can ameliorate memory deficits in murine models without diminishing toxic protein accumulation [11]. Consequently, preventing neuronal loss while restoring function holds substantial promise as a therapeutic strategy for treating AD-an approach that is gaining traction within emerging modalities aimed at addressing this complex disorder [12, 13].

Although microglia are commonly recognized as the immune sentinels of the brain, emerging evidence indicates that they also function as housekeeping cells for neurons, playing crucial roles in their development, functionality, and pathology. The most effective and rapid communication between microglia and neurons occurs through tight membrane-to-membrane contacts. Microglia interact with neuronal cell bodies, synapses, dendrites, and axon initial segments in various ways to support compartment-specific neuronal functions. Additionally, microglia and neurons can engage in long-distance communication *via* intermediate cells and soluble factors. Astrocytes, oligodendrocytes, pericytes, endothelial cells of the neurovascular unit, and peripheral immune cells infiltrating the central nervous system contribute to the intricate remote bidirectional information transmission between microglia and neurons. The interaction mediated by soluble factors such as neuromodulators and cytokines is less specific but more widespread. Microglia can promptly sense changes in neuronal circuit activity through these pathways and subsequently adjust their functional status to provide feedback regulation [14, 15].

Due to the close interactions between microglia and neurons, there is an increasing scientific interest in the role of microglia in the neuron degeneration of AD. We first reviewed how microglia mediate aberrant synaptic pruning in AD. Subsequently, we summarized the interplay between microglial and neuronal metabolism in this context. Finally, we examined the role of microglia within the framework of classical hypotheses regarding neuronal pathology associated with AD.

## 2 SEARCH STRATEGY AND SELECTION CRITERIA

References for this review were identified through searches of PubMed for articles published from January 2015, to October 2024, by the use of the following keywords: “Alzheimer’s disease”, “microglia”, “neurons”, “synaptic pruning”, “lipid metabolism”, “glucose metabolism”, “amyloid cascade hypothesis”, “inflammation”, “tau hypothesis” and “metal ion”. Articles resulting from these searches and relevant references cited in those articles were reviewed. Studies that investigated the microglia-neuron interactions in Alzheimer’s disease were included. Studies that focused on unrelated topics or were published in languages other than English were excluded.

## 3 MICROGLIA-MEDIATED SYNAPTIC PRUNING IN AD

The development of the nervous system involves an overproduction of neuronal synapses, followed by selective elimination of redundant synapses and maturation of the surviving connections [16]. Synaptic pruning refers to the process by which infrequently used synapses are removed while the remaining synapses are strengthened and refined, thereby facilitating the proper establishment and maturation of functional neural networks [17]. Neuronal activity plays a pivotal role in this synaptic pruning process. Synapses that fail to synchronize with their neighboring counterparts receive “penalty” signals from synchronous neurons, causing their elimination [18, 19]. Sensory experiences during developmental stages drive alterations in neuronal activity, resulting in the refinement of associated neural circuits [20]. As a crucial aspect of neuronal maturation, human synaptic pruning peaks approximately two years postnatally and continues into adolescence [20]. Growing evidence suggests that synaptic elimination is also essential for learning processes, memory maintenance, and forgetting throughout adulthood [21]. The involvement of synaptic pruning in neurodevelopmental disorders such as autism spectrum disorder, schizophrenia, and epilepsy has been extensively investigated; moreover, its implications in neurodegenerative diseases like AD, Parkinson's disease, and Huntington's disease are gradually being clarified [16].

The synaptic pruning mediated by microglia is crucial for maintaining homeostasis in the central nervous system. This process is complex and regulated by various factors, including sensory experiences, cytokines, and alcohol exposure [22-25]. On the one hand, microglia recognize and phagocytose inactive or abnormal neuronal synapses through surface receptors such as C-X-C chemokine receptor type 3(CXCR3), P2Y6 receptor, and gamma-aminobutyric acid receptors [26-29]. The complement system and the exposure of phosphatidylserine (PS) are critical in recognizing and eliminating synapses by microglia. C1q initiates the classical complement pathway, activating C3 to “tag” weaker synapses. The binding of C3R on microglia with C3 induces microglial activation, leading to the phagocytosis and removal of these “tagged” synapses [30, 31]. PS directly or indirectly binds to G protein-coupled receptor 56, triggering receptor expressed on myeloid cells 2(TREM2), and Mertk in microglia as a signal that triggers their phagocytic activity towards PS-positive synapses [32-34]. On the other hand, the precise negative regulatory mechanism serves as a guarantee that pruning will not be excessive. Neuronal pentraxins, the sushi domain protein SRPX2, the circadian clock protein BMAL1, progranulin, TREM2, and glucocorticoid receptor antagonism were proven to maintain the homeostasis of synapses by restricting C1q activity [35-40]. Additionally, the CD47-SIRPA axis serves as a classic “don't eat me” signal that inhibits phagocytosis by suppressing integrin signaling activation within microglia [41, 42].

**Fig. (1):**
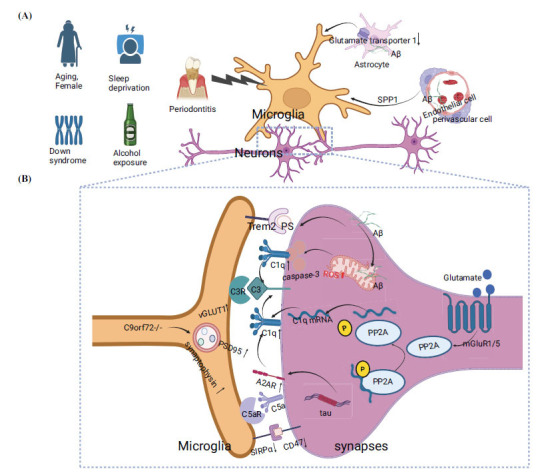
Risk factors and mechanisms of microglia-mediated excessive synaptic pruning in AD. (**A**) Various risk factors associated with AD, including aging, female, Down syndrome, sleep deprivation, periodontitis, and chronic alcohol exposure, have been shown to activate microglia and enhance abnormal synaptic pruning. Additionally, astrocytes and perivascular cells can facilitate microglial synaptic phagocytosis in response to Aβ stimulation. (**B**) The activation of mGluRs, local apoptotic-like mechanisms resulting from mitochondrial damage, and the upregulation of neuronal A2A receptors all contribute to the deposition of synaptic C1q. This process activates complement component C3 to “label” the synapse, thereby promoting microglial activation and enhancing their phagocytic activity towards synapses. Furthermore, the activation of the C5a-C5aR pathway and PS-TREM2 signaling—alongside a deficiency of C9orf72 in microglia—can trigger excessive synaptic pruning and contribute to synaptic loss in AD. Moreover, the CD47-SIRPα axis—which is known for its negative regulation on synaptic pruning—is inhibited in AD. Created with BioRender.com.

The abnormal activation of microglia and their excessive synaptic pruning play a pivotal role in synaptic loss and cognitive impairment in AD [43]. Age and gender are significant risk factors for AD. Research has indicated that the complement components in the brain related to synaptic pruning increase with age, with a more pronounced rise observed in females [44]. Additionally, conditions such as Down syndrome, sleep deprivation, periodontitis, and chronic alcohol exposure—potential contributors to AD—have been shown to activate microglia and enhance abnormal synaptic pruning (Fig. **1A**) [23, 45-47].

Complement-dependent synaptic pruning represents a critical mechanism underlying the excessive elimination of synapses and cognitive impairments in AD [48]. Aβ oligomers upregulate C1q, which subsequently deposits at synapses and activates microglia, leading to early synaptic loss in AD [43]. Mitochondrial dysfunction and septin-related alterations induced by Aβ accumulation may activate local apoptotic-like mechanisms, resulting in the subsequent deposition of synaptic C1q [49]. The deposition of Aβ within blood vessels may indirectly stimulate complement activation and promote microglial phagocytosis of synapses by inducing the secretion of phosphoprotein 1 from perivascular macrophages and fibroblasts. Besides, the astrocytes and neurons are the accomplices in activating of the microglial complement system [50]. Astrocytic glutamate transporter 1 (GLT1) appears to modulate microglial phagocytosis mediated by synaptic C1q. This modulation may be attributed to astrocytic GLT1's role in preventing extracellular glutamate accumulation and subsequent overactivation of metabolic glutamate receptors (mGluRs) [51]. In a tauopathy mouse model, upregulation of neuronal A2AR correlates with increased levels of hippocampal C1q. Within this context, microglial phagocytosis targeting inhibitory synapses is dependent on C1q; deletion of C1q alleviates neurodegeneration and alters the synaptic proteome within tauopathy models [52-54].

The complement-dependent phagocytosis of glutamatergic neurons by microglia necessitates the activation of mGluRs. Evidence has shown that the activation of mGluR1-protein phosphatase 2A (PP2A) signaling in AD facilitates the dephosphorylation of fragile X mental retardation protein, which subsequently leads to the dissociation and synaptic upregulation of C1q mRNA [55]. Inhibition of mGluR1 or blockade of PP2A has been demonstrated to suppress the upregulation of synaptic C1q, resulting in a corresponding reduction in the phagocytosis of glutamatergic synapses by microglia [56]. Furthermore, silencing mGluR5 may confer neuroprotective benefits by hindering the synaptic accumulation of C1q in AD mouse models [57].

Neuroinflammation represents another significant trigger for complement activation in AD. Observations indicate that inflammatory microglia deficient in C9orf72 induce early synapse loss and exacerbate learning and memory deficits through complement-mediated synaptic pruning [58]. In an *in vitro* system simulating AD-related neuroinflammation, interactions between microglia and astrocytes lead to mutual signal transduction processes that result in excessive production of C3 [59]. Additionally, C5a—a pro-inflammatory cleavage product—activates microglia and promotes synapse elimination *via* its receptor, C5aR. The inhibition of the C5a-C5aR signaling pathway has been shown to mitigate AD pathology effectively [60].

In addition to the previously discussed activation of the complement system, other abnormal synaptic pruning mechanisms in AD also warrant attention. Aβ oligomers promote the exposure of PS at synapses, which can be selectively phagocytosed by microglia in a TREM2-dependent manner [61]. The CD47-SIRPα axis functions as a regulatory mechanism to prevent excessive synaptic pruning. In AD, reduced SIRPα levels in microglia result in a failure of this synaptic pruning brake [62].

Abnormal synaptic pruning mediated by microglia not only exacerbates the progression of AD but also contributes to repeated failures observed in clinical trials for AD immunotherapy. Therapeutic antibodies targeting Aβ have been shown to activate complement components that induce the engulfment of synapses by microglia within the brain. Notably, administration of anti-Aβ antibodies lacking the Fc fragment or blockade of CR3 or FcγRIIb did not produce the adverse effects typically associated with these treatments [63]. This finding is anticipated to address some challenges currently hindering progress in AD immunotherapy.

To sum up, microglia initiated excessive phagocytosis of synapses under multiple stresses, which directly resulted in the loss of synapses in AD (Fig. **1B**). The abnormal synaptic pruning mediated by microglia is also a side effect of Aβ immunotherapy, causing repeated failures of its clinical trials. Suppressing this process is not only expected to halt the progression of AD but also enhance the therapeutic effect of AD immunotherapy.

## 4 METABOLIC COUPLING BETWEEN MICROGLIA AND NEURONS IN AD

Despite constituting only 2% of total body mass, the brain accounts for approximately 20% of overall metabolic energy consumption, making it one of the most energy-demanding organs in the body [64]. The signaling processes within neurons—including synaptic transmission, action potential propagation, and maintenance of resting potentials—are particularly energy-intensive [65]. Neuronal activity not only consumes adenosine triphosphate (ATP) but also stimulates its synthesis. The elevation of Rheb induced by neuronal activity can promote the production of acetyl-CoA and ATP in a mammalian target of rapamycin (mTOR)-independent manner while simultaneously regulating mitochondrial gene expression and dynamics through mTOR activation [66]. Glucose is the brain's primary energy substrate, supporting around 95% of ATP production [67]. Typically, mitochondria at neuronal terminals utilize glucose to meet high-energy demands *via* oxidative phosphorylation (OXPHOS), which is a more efficient method for ATP generation. However, to mitigate neurotoxic effects caused by reactive oxygen species (ROS) resulting from electron leakage during OXPHOS, neuronal cytosol shifts towards glycolysis for glucose metabolism—a faster means of producing ATP [68]. In response to stimulation, neurons enhance glucose metabolism while concurrently catabolizing inosine through purine nucleotide phosphorylase; this enables rapid adaptation to fluctuations in neuronal metabolic needs [69]. Branched-chain amino acids represent another crucial substrate for neuronal ATP production, particularly during the perinatal period. The inhibition of SLC7A5, an essential transporter for large neutral amino acids, leads to decreased neuronal excitability, a reduction in neurite number, and behavioral deficits. Mitochondria serve as the primary site for energy substrate consumption in neurons, facilitating energy production and playing a critical role in neuronal development. Enhancing mitochondrial metabolism promotes both morphological and functional maturation of neurons. Conversely, mitochondrial dysfunction adversely affects neuronal morphology and synaptic transmission. For instance, chronic stress-induced mitochondrial fission results in spinal loss and impaired excitatory synaptic transmission; these effects can be mitigated through pharmacological or genetic inhibition of Drp1 [70, 71].

As supporting cells of neurons, the shifts in neurotoxic and neuroprotective phenotypes of microglia arise from their metabolic variability [72]. Microglia exhibit flexible metabolism by utilizing various metabolic substrates depending on nutrient availability and specific functional requirements [73]. The selective inhibition of mitochondrial respiratory complexes due to an imbalance between itaconic acid and nitric oxide (NO) plays a crucial role in regulating microglial reactivity and neurotoxicity. Depletion of microglia or supplementation with itaconate has been shown to be effective for neuroprotection, as evidenced by the preservation of cytoarchitecture and electrical network activity [74]. TREM2 in microglia is essential for the development and metabolic fitness of CA1 neurons. TREM2 deficiency leads to impaired energy metabolism in developing CA1 neurons, which is accompanied by mitochondrial defects and ultrastructural abnormalities in organelles [75]. Furthermore, lysosomal dysfunction and defects in lipid metabolism were observed in TREM2 mutant microglia, resulting in compromised myelin debris degradation [76]. Studies on the demyelination process indicate that TREM2 deficiency causes defects in both cholesterol efflux from microglia and the conversion to disease-associated macrophages (DAM). Subsequent neuronal damage highlights a connection between microglial lipid metabolism and neuronal health [77]. On one hand, microglia can uptake harmful free fatty acids and subsequently release them as lactate and anaplerotic substrates, thereby providing essential energy sources for neurons [78]. On the other hand, disruptions in microglial lipid metabolism have been shown to lead to the accumulation of extracellular lipids, which activate potassium ion channels in neuronal cell membranes. This activation inhibits neuronal firing and reduces neuronal excitability [79]. The metabolic reprogramming of microglia from OXPHOS to glycolysis has emerged as a significant area of research concerning the regulation of microglial functional status. An increase in aerobic glycolysis within microglia is associated with an enhanced inflammatory response and greater neuronal damage [80]. Conversely, inhibiting glycolysis can eliminate microglial activation by suppressing nuclear factor kappa-B (NF-κB) transcriptional activity, ultimately reversing inflammatory-induced neuronal death [81]. Microglial phagocytosis is also energy-intensive and is influenced by mitochondrial function and glucose metabolism. Mitochondrial voltage-sensitive dyes have been employed to isolate microglia exhibiting different metabolic profiles; results indicate that those with high mitochondrial activity demonstrate enhanced phagocytic capacity and transcriptional activation. In line with this finding, the IL33/ST2 axis plays a crucial role in synaptic phagocytosis during brain development by increasing glucose uptake in microglia while enhancing mitochondrial OXPHOS efficiency [82].

**Fig. (2):**
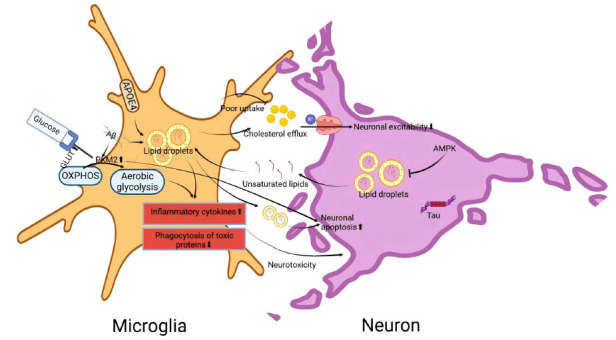
Metabolic coupling between microglia and neurons in AD. The dysregulation of lipid metabolism and the metabolic reprogramming of glucose represent key metabolic characteristics of microglia in AD. The accumulation of lipid droplets in microglia can be attributed to either a disruption in their lipid metabolism or an influx from lipid-overloaded tauopathy neurons. The increase in glucose transporter activity and limiting enzyme activity of glycolysis leads to increased glucose uptake by microglia and an increase in glycolytic activity. Dysregulated glucose and lipid metabolism in microglia stimulates the production of inflammatory factors and inhibits the clearance of toxic proteins, leading to neurotoxicity. Abnormal metabolites and metabolic enzymes can also directly inhibit neuronal excitability, leading to neuronal apoptosis. Created with BioRender.com.

The preceding discussion underscored that microglia metabolism can influence neuronal function through various mechanisms. These processes have been recognized as contributing factors to the neuropathology of AD (Fig. **2**). Human genome-wide association studies reveal a close relationship between lipid-processing microglia and inflammatory microglia, both of which exhibit significant enrichment in AD pathology [83]. Furthermore, studies using App knock-in mouse models have demonstrated that microglia containing Aβ express genes highly enriched for lipid clearance and metabolism, alongside notable changes in lipid metabolites [84]. Interestingly, the lipid metabolism of microglia and neurons is intricately interconnected. Specifically, the accumulation of lipid droplets within microglia observed in tauopathy models may originate from lipid-overloaded tauopathy neurons [85]. Additionally, neuronal conditioned media can stimulate triglyceride synthesis and promote lipid accumulation in apolipoprotein E4 (APOE4) microglia, switching them to an inflammatory phenotype. Dysfunctional lipid metabolism in microglia not only leads to the upregulation of NF-κB-related pro-inflammatory cytokine genes and the secretion of inflammatory chemokines, which directly damages neurons, but also manifests more extensive neurotoxic effects [86]. Specifically, lipid droplet accumulation results in a decreased motility of microglial processes, impairing their capacity to monitor neuronal activity and respond swiftly to neuronal damage. Simultaneously, dysfunctional lipometabolism reduces the efficacy with which microglia uptake extracellular cholesterol; excess cholesterol enhances the expression of G-protein-gated inwardly rectifying K^+^ channel (GIRK3), promoting potassium ion influx and inducing hyperpolarization of the resting membrane potential. This sequence ultimately inhibits neuronal excitability and disrupts normal neural circuit functionality [79]. Of particular concern is that the conditioned medium derived from APOE4 genotype-associated microglial cultures-characterized by high concentrations of lipid droplets-can trigger caspase activation, accelerating the process of neuronal apoptosis [86].

In addition to dysregulation within lipid metabolism pathways, proteomic analyses conducted on microglia from mice with Aβ deposits reveal an upregulation of protein glycosylation and carbohydrate metabolism processes [87]. Activated microglia observed in AD mouse models demonstrate increased glucose uptake; this heightened glucose consumption is further supported by evidence indicating that depletion of microglia can prevent elevated glucose uptake observed in these models [83]. Proteomic analyses performed on AD patients at various stages have shown that protein network modules related to glucose metabolism are significantly associated with AD pathology as well as cognitive dysfunction. These proteins were enriched within microglial populations and present in cerebrospinal fluid during the early stages of AD, suggesting their potential roles as drivers for AD pathogenesis. Interestingly, disorders related to glucose metabolism proteins progressively increase with aging, highlighting the biological basis of aging as a significant risk factor for AD [88]. The observed regional positive association between TSPO-PET and FDG-PET in AD patients further underscores a robust correlation between microglial activity and glucose uptake in the human brain [89]. A study focusing on early-onset AD patients revealed that microglial activation and dysregulation of glucose metabolism co-occur in typical neurodegenerative regions, thereby disrupting frontal connectivity. The glucose ingested in large quantities by AD microglia is metabolized depending on the glycolytic pathway [90]. Prolonged elevation of glycolysis *via* the glycolysis/H4K12la/PKM2 positive feedback loop facilitates the release of inflammatory cytokines and reactive ROS from microglia, resulting in neurotoxic effects [91, 92].

## 5 MICROGLIA-NEURON INTERACTION IN AD HYPOTHESIS

### 5.1 Microglia-neuron Interaction in the Amyloid Cascade Hypothesis

The amyloid cascade hypothesis is a well-established theory regarding the pathogenesis of AD, first proposed three decades ago [93]. This hypothesis posits that amyloid deposition serves as the central event in the development of AD, which can directly lead to neurofibrillary tangles, neuronal loss, and dementia [93, 94]. Aβ is a hydrolysis product derived from amyloid precursor proteins (APPs), catalyzed by β- and γ-secretase enzymes. Factors such as peptide chain length, concentration, solubility, and aggregation state determine whether Aβ exerts beneficial effects on neurons. Within physiological concentration ranges, Aβ is advantageous for synaptic structure and function; however, excessive accumulation can result in neurotoxicity [95, 96]. Aqueously soluble oligomers of Aβ peptides are now recognized as the primary neurotoxic form associated with AD [96]. Aβ disrupts membrane integrity by inserting itself into cell membranes to form ion channels [97]. Additionally, ROS generation and lipid peroxidation induced by Aβ further contribute to damage to neuronal membrane structures [98]. Furthermore, Aβ exerts its cytotoxic effects through interactions with membrane proteins such as PrPc, integrins, and N-methyl-D-aspartic acid receptor (NMDA) [99-101]. Impaired cell membrane integrity and altered permeability facilitate excessive Ca^2+^ influx. It has also been observed that Aβ induces calcium imbalances within organelles such as mitochondria and the endoplasmic reticulum (ER) [102, 103]. Mitochondria play a crucial role in buffering calcium levels; thus, excess Ca^2+^ influx into these organelles impairs oxidative phosphorylation processes while inducing ROS production [104]. This disruption opens mitochondrial permeability transition pores, ultimately leading to neuronal death [104]. The leakage of Ca^2+^ from the endoplasmic reticulum can regulate synaptic plasticity and induce cognitive impairment [105]. Aβ promotes calcium leakage by stimulating the opening of endoplasmic reticulum calcium release channels, which may result in neuronal apoptosis [103]. Furthermore, it has been demonstrated that Aβ interacts with various intracellular structures to exert neurotoxicity. Specifically, Aβ located on telomeres binds to RNA·DNA hybrids, inhibiting telomerase activity and leading to telomere loss and neuronalaging [106, 107]. Notably, Aβ selectively accumulates in poorly acidified autolysosomes within neurons; this is followed by increased lysosomal membrane permeability, release of cathepsins, and subsequent lysosomal cell death [108].

**Fig. (3):**
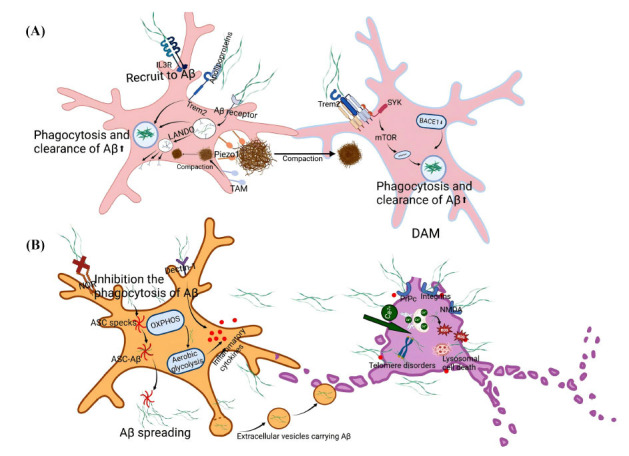
Microglia-neuron interaction in the amyloid cascade hypothesis. (**A**) Microglia exert neuroprotective effects through the clearance of Aβ or the compaction of plaques. The IL-3R on microglia can recruit microglia to aggregate around the Aβ. Various factors, including LC3-related endocytosis (LANDO), the transformation of disease-associated microglia, BACE1 deficiency, and mTOR signaling activation, can significantly augment the phagocytosis of Aβ by microglia. Besides, the Piezo1 and TAM on microglia can compact the Aβ plaques and prevent their dispersion. (**B**) Microglia exhibit neurotoxic effects by promoting Aβ diffusion and reducing its phagocytosis. The NOR on microglia can inhibit the phagocytosis of Aβ. The formation of ASC-Aβ complexes and extracellular vesicles carrying Aβ facilitates the spread of Aβ. Besides, the activation of Dectin-1 and metabolic reprogramming resulting from Aβ stimulation lead to the release of inflammatory factors by microglia. Diffused Aβ can bind to membrane proteins of neurons or enter neurons, thereby damaging the structure of neuronal membranes, inducing calcium overload, causing telomere disorders, impairing the permeability of lysosomal membranes, and eventually resulting in neuronal death. Created with BioRender.com.

Microglia play a crucial role in neuronal protection through the processes of phagocytosis and compaction of Aβ plaque (Fig. **3A**). Stimulation of interleukin-3 receptor (IL-3R) on microglia induces both transcriptional and functional reprogramming, resulting in the aggregation of microglia around Aβ deposits and facilitating Aβ clearance [109]. The LC3-associated endocytosis (LANDO) pathway and Nogo receptor (NgR) are also vital for modulating the phagocytic activity of microglia towards Aβ. Specifically, LANDO enhances Aβ clearance by promoting the recycling of Aβ receptors and increasing their surface expression [110]. Conversely, the expression of NgR on microglia impairs their ability to phagocytose and clear Aβ [111]. DAM represent a specific phenotype that aggregates around Aβ plaques, enabling effective phagocytosis of these aggregates [112]. TREM2 and SYK are key regulatory factors that facilitate the transition from resting to DAM phenotypes while also regulating microglial phagocytosis [111, 113, 114]. Notably, Aβ can bind to apolipoproteins that serve as ligands for TREM2, thereby enhancing its uptake by microglia in a TREM2-dependent manner [115]. The absence of β-Site APP-Cleaving Enzyme (BACE1) or activation of mTOR signaling within microglia promotes an upregulation of genes associated with phagocytic activity, further aiding in the clearance of Aβ [116, 117]. Dense aggregates of Aβ restrict neuronal exposure to diffuse toxic Aβ; however, the formation of dense Aβ plaques does not occur spontaneously. Microglial Piezo1 senses mechanical stiffness induced by Aβ fibrils and regulates plaque compression accordingly [118]. Additionally, TAM receptors on microglia facilitate the transport of engulfed Aβ into acidic lysosomes where it is compacted into dense plaques [119].

Aβ activates microglia, prompting the release of substantial amounts of inflammatory cytokines, which contributes to neurotoxicity (Fig. **3B**) [120]. The pro-inflammatory activation of microglia is characterized by a metabolic shift from OXPHOS to glycolysis. Lactate-dependent histone modifications in microglia adjacent to Aβ plaques are enriched at the promoters of glycolytic genes, thereby enhancing transcription and increasing glycolytic activity [92]. Exosomes containing Aβ derived from neurons induce metabolic reprogramming in microglia, which can sustain a neuroinflammatory environment [121]. Furthermore, Aβ can directly bind to Dectin-1 on microglia, stimulating their activation and the expression of inflammatory factors. Knockdown of Dectin-1 has been shown to reduce Aβ-induced microglial activation, inflammatory responses, synaptic loss, and cognitive deficits [122]. Notably, the deposition of Aβ leads to the formation of apoptosis-associated speck-like protein containing caspase recruitment domain (ASC) specks within microglia that are dependent on inflammatory vesicles. ASC rapidly associates with Aβ, facilitating the propagation of Aβ pathology. Compounding this issue is that ASC-Aβ complexes further promote nucleotide-binding oligomerization domain-like receptor protein 3 (NLRP3) activation in neighboring microglia while diminishing their capacity to clear Aβ effectively [123, 124]. In addition to these indirect neurotoxic effects described above, large extracellular vesicles carrying Aβ secreted by microglia directly alter dendritic spine morphology and impair synaptic plasticity within the entorhinal cortex-dentate gyrus circuitry [125].

### 5.2 Microglia-neuron Interaction in the Tau Hypothesis

The formation of neurofibrillary tangles (NFTs) has been recognized as a pathological hallmark of AD since its initial description in 1907. NFTs are primarily composed of paired helical filaments formed by aggregates of hyperphosphorylated tau protein [126]. All six isomers of tau are present within NFTs, and the cross-seeding among these tau isomers facilitates NFT growth [127]. Tau is a highly soluble protein that binds to microtubules, thereby promoting their assembly and stability. Various post-translational modifications—including phosphorylation, glycosylation, acetylation, and truncation—interact to enhance tau aggregation. Among these modifications, phosphorylation stands out as the most prevalent and extensively studied alteration. The interactome associated with phosphorylated tau(p-tau) in NFTs is enriched for proteins involved in the ubiquitin-proteasome and phagosome-lysosome pathways, suggesting that toxic accumulation of tau correlates with impairment in protein degradation mechanisms [128]. The negative correlation between p-tau levels and synaptic health and density has raised increasing concerns about the tau protein hypothesis. Several large-scale clinical studies have demonstrated that plasma p-tau217, p-tau181, and p-tau231 serve as effective biomarkers for distinguishing AD due to their non-invasive nature and ease of accessibility [129-133]. Imaging studies utilizing tau tracers indicate that regions exhibiting longitudinal accumulation of tau correspond to Braak stages; as clinical symptoms progress, the primary area of tau deposition shifts from the medial temporal lobe to the sensorimotor cortex [134]. Concurrently, levels of tau-related molecules in cerebrospinal fluid and plasma gradually increase throughout the progression of AD. A study integrating both cross-sectional and longitudinal cohorts reveals that microtubule-binding region (MTBR)-tau-243 can effectively differentiate between various clinical stages of AD—a finding corroborated by results from positron emission tomography (PET) imaging targeting tau pathology [135].

The detrimental impact of pathogenic tau on synaptic plasticity and neuronal structure is a primary factor contributing to the positive correlation between tau levels and cognitive impairment [11]. Tau mislocalizes to presynaptic terminals, thereby restricting the mobility of synaptic vesicles and resulting in defects in neurotransmitter release [136]. The deposition of tau in the ventral hippocampus significantly diminishes the excitability of ventral hippocampal neurons while suppressing their distinct firing patterns associated with discrimination tasks [137]. Furthermore, disruptions in theta synchronization between the medial septum (MS) and CA1 subsets during memory consolidation highlight the neurotoxic effects of tau accumulation within the MS-hippocampal CA1 cholinergic circuit [138]. Neuronal nuclear envelopes and chromatin are particularly susceptible to pathogenic tau attacks, which are accompanied by alterations in gene expression. Extracellular tau oligomers (xcTauO) synergize with intracellular tau to exert neurotoxicity by inducing invagination of neuronal nuclei and disrupting nucleoplasmic transport. Notably, xcTauO promotes histone methylation and upregulates the MAPT gene encoding for tau, thus driving increased production of toxic forms of this protein [139]. In models of Drosophila tauopathy, nuclear invagination induced by tau occurs without a critical RNA quality control mechanism—the nonsense-mediated mRNA decay pathway—resulting in an accumulation of proteins encoded by defective RNAs within Drosophila brains [140]. Additionally, heterochromatin decondensation and dysregulation of transposable elements caused by pathological tau lead to elevated levels of adult neuroinflammatory double-stranded RNA (dsRNA) as well as neuronal death [141, 142]. Imbalance in calcium homeostasis is an undeniable contributor to tau neurotoxicity. Prior to neuronal loss, the tau mutation significantly enhances the excitability of the basal network, which can be attributed to increased binding of CaMKII and decreased binding to calmodulin and neurogranule proteins [143]. Elevated calcium leakage is associated with tau accumulation during aging. Inhibition of calcium leakage has been shown to improve neuronal firing and cognitive function [144]. Furthermore, p-tau induces neuronal death through necroptosis and inflammation, mediated by the formation of the RIPK1/RIPK3/MLKL necrosome and activation of NF-κB pathways [145, 146].

**Fig. (4):**
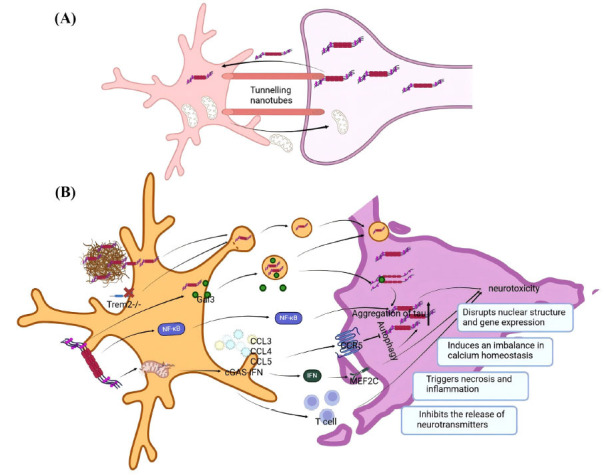
Microglia-neuron interaction in the tau hypothesis. (**A**) Microglia can transfer and clear neuronal tau aggregates through tunneling nanotubes and deliver healthy mitochondria to affected neurons. (**B**) Microglia exert neurotoxic effects by promoting the seeding and accumulation of tau aggregates. Tau aggregates phagocytosed by microglia can be delivered to neurons *via* extracellular vesicles. The release of Gal3 or a deficiency of TREM2 can exacerbate this process. The free Gal3 and NF-κB generated by tau-stimulated microglia can promote the formation and accumulation of tau aggregates within neurons. In addition, activated microglia can further inhibit neuronal autophagy by binding to CCR5 receptors *via* chemokines, and subsequently prevent the clearance of tau aggregates. Microglia can also suppress the neuronal MEF2C transcription, recruit T cells to infiltrate the brain parenchyma, and thereby generate neurotoxicity. Created with BioRender.com.

Microglia can rescue tau-burdened neurons by reducing the aggregation of tau and damaged mitochondria (Fig. **4A**). In detail, microglia connect with tau-laden neurons through tunneling nanotubes, facilitating the transfer of these proteins to microglia for subsequent clearance. Additionally, tunneling nanotubes enable the transport of healthy mitochondria from microglia to tau-affected neurons, thereby safeguarding neurons against the detrimental effects of damaged mitochondria [147, 148].

Unfortunately, in most cases, microglia act as accomplices in the progression of tau pathology, promoting the spread and accumulation of tau aggregates (Fig. **4B**). AD2 microglia are predominantly observed in AD samples characterized by tau pathology, indicating an immune response to tauopathy neurons [149]. The enhanced spread of tau pathology within amyloid-beta plaques is attributed to neurodegenerative microglia, as evidenced by their secretion of bulk extracellular vesicles containing tau seeds following the phagocytosis of p-tau-positive aggregates deposited on the plaque [150]. Furthermore, the aggregation of senescent microglia in mice with tau pathology has been shown to promote both tau phosphorylation and the accumulation of insoluble tau [151]. TREM2 knockout mice were utilized to demonstrate that the absence of TREM2 increases the release and seeding potential of microglial exosomal tau, resulting in its dispersion within neurons [152]. Additionally, activation of the NF-κB signaling pathway in microglia by pathological tau represents another critical mechanism driving both diffusion and toxicity associated with this protein [153]. Upon exposure to pathological tau, a subpopulation of microglia releases Galectin-3 (Gal3) in both free and extracellular vesicle-associated forms. The free form of Gal3 can penetrate into tau-laden neurons where it binds directly to p-tau, significantly enhancing tau-fibrosis. Moreover, Gal3-dependent extracellular vesicles are capable of transporting p-tau. In summary, Gal3 released by microglia promotes the accumulation of misfolded tau within recipient neurons [154]. Moreover, the binding of C-C motif chemokine ligand 3/4/5 (CCL3/4/5) to C-C motif chemokine receptor 5 (CCR5) has been identified as a critical mediator facilitating detrimental interactions between microglia and neurons. Research indicates that activated microglia can inhibit neuronal autophagy *via* CCR5 signaling pathways; this impairment hinders the clearance of abnormally aggregated proteins [155].

When abnormal tau exists in microglia or neurons, it will result in abnormal microglia-neuron interactions. The overexpression of “eat me” signals on neurons and synapses during tau pathology accelerates the clearance of synapses by microglia, leading to early impairments in brain function associated with AD [156-158]. Additionally, increased lipogenesis and slow lipid turnover observed in tauopathy-affected neurons result in the accumulation of lipid droplets, which are subsequently transferred to microglia. This aberrant accumulation of lipid droplets within microglia leads to heightened oxidative stress, an increase in pro-inflammatory factor expression, and impaired phagocytic activity [85]. In mouse models exhibiting tauopathy, the activation of cyclic GMP-AMP synthase (cGAS) triggers a type I interferon response in microglia, which suppresses neuronal myocyte enhancer factor 2C (MEF2C) transcription. This suppression ultimately results in synapse loss and memory deficits. Conversely, genetic ablation or pharmacological inhibition of cGAS enhances neuronal MEF2C transcriptional networks, thereby rescuing both synapse loss and cognitive function without affecting tau load [159]. In addition, activated microglia have been shown to recruit T cells into brain parenchyma—contributing further to neurodegeneration [160].

### 5.3 Microglia-neuron Interaction in the Inflammatory Hypothesis

The term “neuroinflammation” refers to the inflammatory response of the central nervous system that occurs following pathological damage [161, 162]. The role of activated microglia and their innate immune responses in neuroinflammation is significant and cannot be overlooked. Neuroinflammation has been identified as a critical feature of AD, which synergistically accelerates AD progression in conjunction with other hypotheses. On the one hand, Aβ, pathological tau, excessive metal ions, and metabolic reprogramming can activate microglia and promote the release of inflammatory cytokines. On the other hand, inflammatory microglia facilitate Aβ pathology and the propagation of pathological tau, reducing the clearance of these toxic proteins. As early as the 1990s, anti-inflammatory treatments were shown to reduce the incidence of AD in several large-scale epidemiological studies. Similar findings have been observed in animal models of AD [163]. Recently, single-nucleus transcriptome analyses combined with *in situ* hybridization have elucidated the status of microglia in patients with AD, revealing a significant increase in inflammatory state microglia among these individuals [83]. Furthermore, another single-nucleus RNA sequencing analysis demonstrated an enhanced profile of the inflammatory endolysosomal network within microglia from AD patients [164]. Additionally, a cross-sectional study encompassing various stages of AD uncovered a robust association between neuroinflammation and a range of neuropsychiatric manifestations, including irritability, agitation, and nocturnal disturbances [165].

The heterogeneity of microglia contributes to their complex role in the neuroinflammation of AD. The traditional classification of microglia into M1 pro-inflammatory and M2 anti-inflammatory phenotypes presents significant limitations. In AD, the activation of microglia is continuous and diverse. Recent advancements in single-cell sequencing have identified subpopulations such as DAM and terminal inflammatory microglia. DAM can be further categorized into pro-inflammatory subtypes characterized by high expression levels of CD44, CD45, and Kv1.3 channels, and anti-inflammatory subtypes exhibiting elevated CXCR4 expression. The regulation of pro-inflammatory subtypes involves pathways such as NFkB, Stat1, and RelA, whereas anti-inflammatory subtypes are regulated by LXRα/β signaling [166]. Additionally, osteopontin production allows for the division of DAM into protective CD11c+OPN- subtypes and CD11c+OPN+ pro-inflammatory subtypes [167]. Terminal inflammatory microglia, a distinct category that differs from DAM, are enriched in AD mice and highly express inflammatory genes to promote neuroinflammation [168]. Despite these insights, numerous microglial subtypes within AD remain unidentified, and the dynamic changes among different subtypes have yet to be fully explored.

What factors influence the microglia-associated neuroinflammation in AD? A study involving 50 participants demonstrated that activated microglia are distributed along functional rather than structural connectivity, indicating that regions of neuroinflammation are likely interconnected through neuronal activity [169]. The genetic predisposition to AD renders the brain more susceptible to neuroinflammation. Inhibition of INPP5D, a gene associated with genetic risk for AD, can induce NLRP3 inflammasome formation and CASP1 cleavage in microglia *in vitro*. Similar findings have been observed in the brains of individuals with AD [170]. In AD patients carrying the APOE allele, excessive activation of pro-inflammatory cytokines—resulting from an imbalance in T-cell immune responses—has been linked to downregulation of the IL-7/IL-7R signaling pathway [171, 172]. Additionally, crosstalk between microglia and the adaptive immune system has been shown to modulate neuroinflammation in other studies. Adaptive immune deficiency can exacerbate microglial activation and inflammatory gene expression in mouse models of AD; conversely, regulatory T-cell treatment has been found to alleviate inflammation and provide neuroprotective effects [173]. In a mouse model of AD, CD8+ T cells serve as a brake on neuroinflammation by co-localizing with microglia and limiting their inflammatory response *via* the CXCL16-CXCR6 axis [174]. However, research utilizing a three-dimensional human neuroimmune axis model suggests that excessive infiltration of peripheral T cells may worsen neuroinflammation in AD. CXCL10 secreted by astrocytes affected by AD binds to CXCR3 on peripheral T cells, recruiting them into the central nervous system and activating the IFN/inflammatory pathway within microglia [175]. The relationship between signaling molecules derived from peripheral organs and the inflammatory state of microglia is consistently regarded as a critical link bridging peripheral immunity with central nervous system processes. Metabolites such as phenylalanine, isoleucine, and polyunsaturated fatty acids, along with neurotransmitters including acetylcholine, glutamate, dopamine, and norepinephrine produced by gut microbiota, drive microglial activation and contribute to neuroinflammation in AD [176-178]. Additionally, adiponectin derived from the liver serves as another important bridge connecting peripheral signals to neuroinflammation. Notably, overexpression of trimeric adiponectin in the livers of AD mice elevates central levels of adiponectin and subsequently inhibits the activation of the NLRP3 inflammasome in microglia [179].

**Fig. (5):**
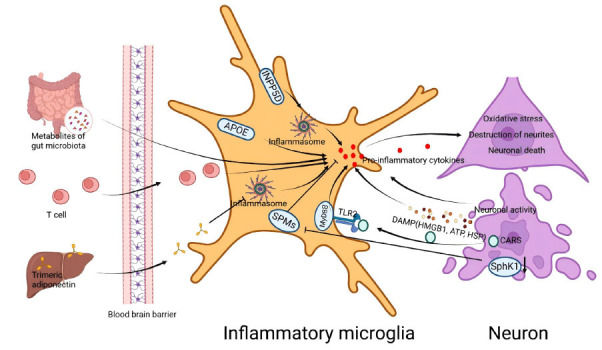
Microglia-neuron interaction in the inflammatory hypothesis. Microglial inflammation may be triggered by disturbances in gut microbial metabolites, decreased levels of trimeric adiponectin in the liver, and central infiltration of peripheral T cells. Genetic risk factors associated with AD, such as INPP5D and APOE, are also closely linked to the activation of microglia. The excessive inflammatory factors released by microglia can lead to neuronal oxidative stress, neurite damage, and even neuronal death. Abnormal neurons in AD, in turn, contribute to the activation of microglial inflammation. Elevated levels of cysteine-tRNA synthetase in neurons and damage-associated molecular patterns released during cell death both contribute to the inflammatory activation of microglia. The absence of SphK1 in neurons also impedes the resolution of microglial inflammation by suppressing the secretion of specialized pro-resolving mediators. Created with BioRender.com.

The neuronal damage induced by microglia-mediated inflammation significantly contributes to the cognitive impairments associated with AD (Fig. **5**). Excessive inflammatory factors, such as IL-1β, interferon-gamma (IFN-γ), and tumor necrosis factor-α (TNF-α), produced by activated microglia can disrupt neurotransmitter transmission and impair synaptic plasticity in neurons. This disruption underlies the learning and memory deficits observed in AD [180]. The transcription factor MEF2c, which serves as a protective regulatory factor for cognitive resilience, can be inhibited by inflammatory signaling from IFN in microglia. Such inhibition results in synaptic damage and cognitive impairment in mouse models exhibiting tau pathology [159]. Neuroinflammation can also lead to the destruction of neurites that connect neural networks; this occurs as an upregulation of superoxide production induces oxidative stress in adjacent neurites. The formation of cofilactin rods and aggregates driven by oxidative stress may result in neurite degeneration without causing immediate neuronal death [181]. However, prolonged inflammation ultimately leads to observable neuronal death over time.

As previously mentioned, neurons are significantly affected by microglial inflammation in AD. Interestingly, neurons also contribute to the activation of microglial inflammation. Proteomic analysis of cerebrospinal fluid from patients with autosomal AD has revealed an enrichment of mitochondrial damage and NMDAR synaptic signaling proteins during the early stages of the disease, neuronal apoptotic proteins in the intermediate stages, and microglia-associated innate immune system proteins in the later stages [182]. This suggests that substantial neuronal death occurs prior to a widespread immune response mediated by microglia. The release of intracellular pro-inflammatory cytokines (*e.g*., IL-1β, IL-6, IL-33, TNF-α) and damage-associated molecular patterns (*e.g*., HMGB1, HSP, ATP) during neuronal death serves as triggers for microglial inflammation [183, 184]. Furthermore, abnormally elevated levels of cytoplasmic cysteinyl-tRNA synthetase in AD neurons activate the TLR2/ MyD88 signaling pathway within microglia, leading to the production of significant amounts of inflammatory cytokines [185]. In addition to promoting microglial inflammation, neurons also impede the resolution of this inflammation by suppressing the secretion of specialized pro-resolving mediators. Sphingosine kinase 1 (SphK1) present in neurons facilitates acetylation of cyclooxygenase-2, which is instrumental in secreting these specialized pro-resolving mediators. However, a deficiency in SphK1 within AD neurons hinders effective resolution of inflammation [186].

### 5.4 Microglia-neuron Interaction in the Metal Ion Hypothesis

The role of iron in the nervous system warrants special attention, as it is a crucial component of myelin and neurotransmitters, and plays a significant role in DNA synthesis and energy metabolism [187]. Ferroptosis is a distinct form of cell death that was proposed in 2012, characterized by intracellular iron accumulation and lipid peroxidation. The dysregulation of iron metabolism results in the intracellular accumulation of Fe^2+^, which catalyzes lipid peroxidation *via* the Fenton reaction. This process leads to damage to the cell membrane and mitochondrial dysfunction, accompanied by the continuous accumulation of lipid peroxidation products, ultimately resulting in cell death [188]. Iron deposition has been observed in the early stage of the AD brain, and its distribution is consistent with the brain regions where neuronal degeneration and brain atrophy occur [189]. The positive correlation between Aβ plaque burden, tau pathology, and iron levels has strengthened the association between iron metabolism disorders and AD [190]. Recent evidence suggests that iron metabolism, microglial activity, and neuropathology are intricately linked in AD. Microglia are particularly vulnerable to iron accumulation due to their capacity to phagocytose damaged myelin debris, which is abundant in both iron and lipids [191]. In patients with AD, numerous microglia laden with iron have been observed surrounding Aβ plaques [192]. The increased presence of ferritin and elevated levels of iron within microglia contribute to a shift towards a glycolytic phenotype while promoting the release of pro-inflammatory cytokines [193]. Furthermore, iron-mediated lipid peroxidation can lead to DNA damage and subsequent microglial death, accompanied by the generation of substantial amounts of reactive oxygen species [191]. In tri-culture systems comprising microglia, astrocytes, and neurons, it has been demonstrated that microglia respond more rapidly to ferroptosis inducers than either astrocytes or neurons; this indicates their heightened susceptibility to ferroptosis [194, 195]. Notably, significant neuronal lipid peroxidation and loss were observed exclusively in the presence of microglia—suggesting that ferroptosis within these cells is responsible for neurotoxicity [196].

Copper homeostasis is essential for proper functioning of the nervous system, as it regulates synaptic activity, neurotransmitter synthesis, and information transmission. The maintenance of copper homeostasis relies on the coordinated regulation of a protein network that includes copper enzymes, copper chaperones, and membrane transporters. Copper is absorbed into the bloodstream through the intestinal copper transporter CTR1, which subsequently binds to soluble chaperones for transportation to specific tissues and organs. Within the cytoplasm, Cu chaperones facilitate the transfer of Cu to the trans-Golgi network, mitochondria, and nucleus to carry out essential physiological functions. In addition, the efflux of copper from cells is dependent on Cu-ATPases ATP7A and ATP7B [197]. Abnormal copper levels have been observed in patients with AD and various animal models, with substantial evidence supporting its role in the progression of AD [198]. The rapid release of copper from neurons is linked to the activation of NMDA receptors; overactivation of these receptors in AD may contribute to elevated copper concentrations within the hippocampus [199]. Excessive copper can bind to Aβ with high affinity, forming a complex that prompts microglia to release TNF-α and NO, thereby exerting neurotoxic effects [200]. Complicating this scenario, another study indicates that microglia can enhance copper uptake, transport, and accumulation under the stimulation of IFN-γ, suggesting a potential neuroprotective mechanism in AD [199]. While both synergistic and protective roles of microglia within the context of copper dysregulation have been documented, it remains unclear how these two aspects are modulated or switched between one another—an area ripe for future research exploration.

Zinc is a trace metal ion obtained from dietary sources, existing in a stable form bound to metalloproteins or in an unstable ionic state. The brain is the organ with the highest concentration of zinc in the human body. Maintaining a normal level of zinc is crucial for both the growth and development of the brain, as well as for effective signal transduction in neurons [201]. Both excessive and insufficient levels of zinc can lead to neurological disorders. The maintenance of zinc homeostasis is governed by three distinct protein families. Metallothioneins play a crucial role in regulating intracellular zinc levels. Zinc and iron-like regulatory proteins facilitate the uptake of zinc into cells and its subsequent intracellular transport. Zinc transporters are responsible for the efflux of zinc from cells [202]. The disruption of zinc homeostasis is associated with the progression of AD, and zinc deficiency has been identified as a risk factor for this condition [203]. A total of 582 zinc-related differentially expressed genes were identified in patients with AD compared to healthy individuals, while 146 zinc-related differentially expressed genes were found in AD mice *versus* normal mice [204]. Zinc deficiency-induced activation of NLRP3 and the subsequent enhancement of inflammatory responses in microglia contribute to neuronal death and cognitive impairment in AD [203]. Supplementation of zinc to inflammatory microglia cultured *in vitro* has been shown to protect the viabilities of co-cultured neurons [205]. In addition, the spherical oligomers formed by the combination of Zn and Aβ can activate microglia and enhance the neurotoxicity of Aβ [206].

## CONCLUSION

As the housekeeping cells of neurons, microglia respond to external stressors and continuously remodel the neuronal microenvironment to shape neuronal fate. Recent discoveries regarding the structure and mechanisms underlying bidirectional communication between microglia and neurons—such as tunnel nanotubes—have significantly enhanced our understanding of the role of microglia in the neuropathology of AD. However, due to the heterogeneity among microglia, the interaction between microglia and neurons in AD cannot be generalized. Specifically, in AD, microglia show excessive phagocytosis of synapses while exhibiting inadequate clearance of toxic aggregated proteins. Both optogenetic depolarization of microglia and Aβ immunotherapy aimed at eliminating Aβ have been shown to enhance the synaptic phagocytic activity of microglia. Avoiding facilitated synaptic phagocytosis during the enhancement of Aβ clearance by microglia can significantly amplify the therapeutic efficacy. Furthermore, microglia exhibit a dual role in the accumulation of toxic proteins. On one hand, they promote the clearance of these toxic proteins, thereby limiting their diffusion and protecting neurons. On the other hand, they also contribute to the formation and dissemination of toxic proteins that ultimately damage neurons. The exploration of new technologies and methodologies for distinguishing and manipulating subtypes of microglia represents a crucial avenue for maximizing neuroprotective effects while mitigating risk factors associated with neuronal damage.

The existing human studies primarily reflect the accumulation of Aβ, tau pathology, microglial activation, and neuronal damage through neuroimaging and biomarker detection. Multi-omics analyses of postmortem brain tissues in humans have unveiled the polymorphism and uniqueness of microglia in patients with AD. Research into the mechanisms of AD has relied heavily on non-human studies due to the difficulty of obtaining human brain tissue. However, species dissimilarities limit the applicability of findings from non-human studies to clinical settings. Therefore, it is essential to refine human surrogate systems such as brain organoids for translating basic research into clinical practice. Current therapeutic strategies focus on the regulation of inflammatory responses and the phagocytosis of toxic proteins by microglia. AL002, an agonistic antibody targeting TREM2, has been demonstrated to activate microglia, thereby promoting their proliferation and enhancing their phagocytosis of Aβ [207]. AN1792, a classic representative for stimulating active immunity in AD, effectively reduces stress-induced reactive microglia and further facilitates the clearance of Aβ [208]. Moreover, optogenetic depolarization of microglia significantly enhances their efficiency in clearing Aβ; however, this process is accompanied by synaptic elimination mediated by complement C1q [209]. Therefore, the combined application of optogenetic depolarization of microglia alongside C1q-blocking antibodies presents a promising approach for improving therapeutic outcomes while minimizing drug-related side effects.

As research on microglia-neuron interaction expands, therapies targeting manipulating microglia to alleviate neuropathology may become a clinical reality. However, the studies reviewed in this article appear to present contradictory findings. For instance, the activation of microglial TREM2 is detrimental in some studies but exerts neuroprotective effects in others. These discrepancies are likely related to variations in the pathological stages of AD, regional specificity within the brain, and genetic polymorphisms of TREM2. Furthermore, the contradictions observed regarding microglial depletion may stem from its heterogeneity that has not yet been thoroughly investigated. Despite the widely acknowledged complexity and diversity of microglial-neuronal interactions, current research has predominantly concentrated on a single way. Exploring the correlations among the diverse mechanisms involved in microglial-neuron interaction constitutes a critical focus for future studies. Discovering commonalities between distinct mechanisms or formulating combination therapies targeting multiple pathways may serve as an essential direction for breaking through the present therapeutic bottleneck.

## LIST OF ABBREVIATIONS

AD: Alzheimer’s Disease
APOE: Apolipoprotein E
ASC: Apoptosis-associated Speck-like Protein Containing Caspase Recruitment Domain
ATP: Adenosine Triphosphate
Aβ: Amyloid β
BACE1: β-Site APP-cleaving Enzyme
CCL3/4/5: C-C Motif Chemokine Ligand 3/4/5
CCR5: C-C Motif Chemokine Receptor 5
cGAS: GMP-AMP Synthase
CXCR3: C-X-C Chemokine Receptor Type 3
DAM: Disease-associated Macrophages
dsRNA: Double-stranded RNA
ER: Endoplasmic Reticulum
Gal3: Galectin-3
GLT1: Glutamate Transporter 1
IFN: Interferon
IL: Interleukin
LANDO: LC3-associated Endocytosis
MEF2C: Myocyte Enhancer Factor 2C
mGluRs: Metabotropic Glutamate Receptors
MS: Medial Septum
mTOR: Mammalian Target of Rapamycin
NF-κB: Nuclear Factor Kappa-B
NFTs: Neurofibrillary Tangles
NgR: Nogo Receptor
NLRP3: Nucleotide-binding Oligomerization Domain-like Receptor Protein 3
NMDA: N-methyl-D-aspartic Acid Receptor
NO: Nitric Oxide
OXPHOS: Oxidative Phosphorylation
p-tau: Phosphorylated Tau
PET: Positron Emission Tomography
PP2A: Protein Phosphatase 2A
PS: Phosphatidylserine
ROS: Reactive Oxygen Species
SIRPA: Signal Regulatory Protein Alpha
SphK1: Sphingosine Kinase 1
TAM: Tyro3/Axl/Mer
TNF-α: Tumor Necrosis Factor-α
TREM2: Triggering Receptor Expressed on Myeloid Cells 2
xcTauO: Extracellular Tau Oligomers
